# Cancer-related transcription regulator protein NAC1 forms a protein complex with CARM1 for ovarian cancer progression

**DOI:** 10.18632/oncotarget.25400

**Published:** 2018-06-19

**Authors:** Naomi Nakayama, Gyosuke Sakashita, Yuko Nariai, Hiroaki Kato, Kaori Sinmyozu, Jun-ichi Nakayama, Satoru Kyo, Takeshi Urano, Kentaro Nakayama

**Affiliations:** ^1^ Department of Biochemistry, Shimane University School of Medicine, Izumo, Japan; ^2^ Department of Obstetrics and Gynecology, Shimane University School of Medicine, Izumo, Japan; ^3^ Proteomics Support Unit, RIKEN Center for Developmental Biology, Kobe, Japan; ^4^ Graduate School of Natural Sciences, Nagoya City University, Nagoya, Japan; ^5^ Current address: National Cerebral and Cardiovascular Center, Osaka, Japan

**Keywords:** nucleus accumbens-associated protein 1 (NAC1), coactivator-associated arginine methyltransferase 1 (CARM1), protein arginine N-methyltransferase 4 (PRMT4), ovarian cancer

## Abstract

NAC1 is a cancer-related transcription regulator protein that is overexpressed in various carcinomas, including ovarian, cervical, breast, and pancreatic carcinomas. NAC1 knock-down was previously shown to result in the apoptosis of ovarian cancer cell lines and to rescue their sensitivity to chemotherapy, suggesting that NAC1 may be a potential therapeutic target, but protein complex formation of intranuclear NAC1 in ovarian cancer cells remain poorly understood. In this study, analysis of ovarian cancer cell lysates by fast protein liquid chromatography on a sizing column showed that the NAC1 peak corresponded to an apparent molecular mass of 300–500 kDa, which is larger than the estimated molecular mass (58 kDa) of the protein. Liquid chromatography-tandem mass spectrometry analysis identified CARM1 as interacting with NAC1 in the protein complex. Furthermore, tissue microarray analysis revealed a significant correlation between CARM1 and NAC1 expression levels. Ovarian cancer patients expressing high levels of NAC1 and CARM1 exhibited poor prognosis after adjuvant chemotherapy. Collectively, our results demonstrate that high expression levels of NAC1 and its novel binding partner CARM1 may serve as an informative prognostic biomarker for predicting resistance to chemotherapy for ovarian cancer.

## INTRODUCTION

Ovarian cancer is the most aggressive gynecological malignancy worldwide [1] and its incidence has markedly increased in the last decade. In more than 70% of cases, tumors have disseminated beyond the ovaries at the time of diagnosis and treatment of these cases requires combined surgery and chemotherapy. First-line chemotherapy with platinum and taxanes drugs yields a response rate of more than 80%; however, nearly all patients relapse. Recurrent cancers are frequently resistant to platinum and, in most patients, are fatal. Therefore, drugs targeting specific molecular pathways that regulate either metastasis or relapse, or agents targeting pathways altered in chemoresistant tumors, may greatly benefit patients with this disease. A dualistic model of ovarian carcinogenesis has been proposed and number of molecular and histopathological studies were published to provide insight into molecular pathogenesis of ovarian cancer [2]. A clearer understanding of the molecular pathways and genetic alterations underlying ovarian carcinogenesis is therefore a prerequisite to designing these specific chemotherapeutic agents [3, 4].

Nucleus accumbens-associated protein 1 (NAC1), encoded by the *NACC1* gene, is a nuclear protein that encompasses an N-terminal BTB/POZ (broad complex, tramtrack, bric-a-brac /poxvirus and zinc finger) (hereafter abbreviated BTB) and a C-terminal BEN (BANP, E5R and NAC1) domain. The BTB domain is a ∼100 amino acid highly conserved motif that mediates homodimerization and/or heterodimerization and interacts with other proteins [5, 6]. NAC1 homodimerizes through its BTB domain [7] and heterodimerizes with Myc-interacting zinc-finger protein 1 (Miz1) through the respective BTB domain [8, 9]. NAC1 lacks characteristic DNA-binding domains but instead contains a C-terminal BEN domain. Computational analysis has identified BEN domain and suggested that the domain mediates protein-DNA and protein-protein interactions [10].

NAC1 was originally identified and cloned as a cocaine-inducible transcript from the nucleus accumbens, a unique forebrain structure involved in reward motivation and addictive behavior [11]. *NACC1* was also identified as a cancer-associated BTB gene by serial analysis of gene expression in ovarian cancer cells [12]. NAC1 is significantly overexpressed in several types of carcinomas, including ovarian, colorectal, breast, renal cell, cervical, and pancreatic carcinomas, is associated with tumor growth and survival, and increases the resistance of tumor cells to chemotherapy [12–22]. These reports suggested that NAC1 plays various functional roles in cancer development and that it might be a potential therapeutic target.

## RESULTS

### Coactivator-associated arginine methyltransferase 1 (CARM1) interacts with NAC1 in cancer cells

**Figure 1:**
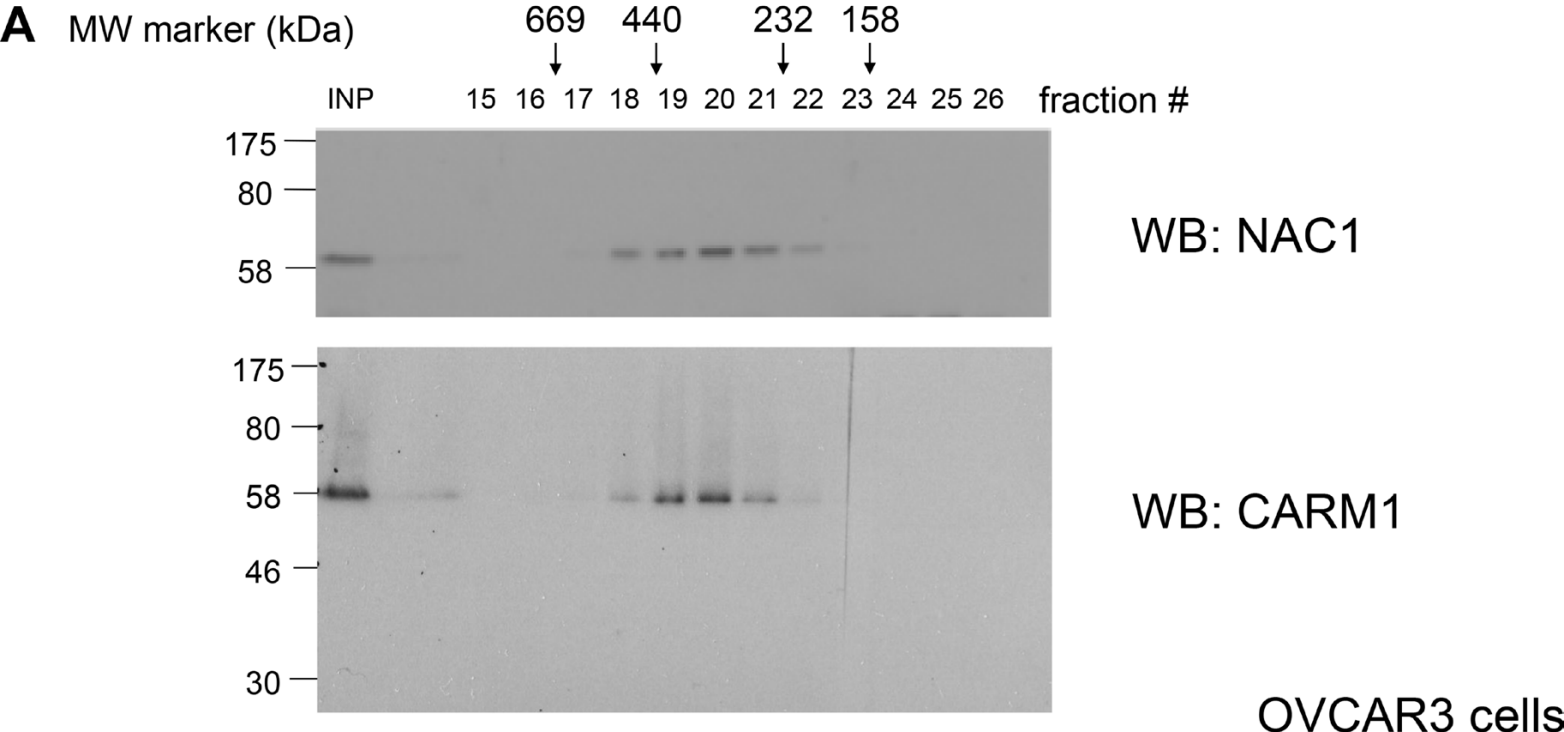

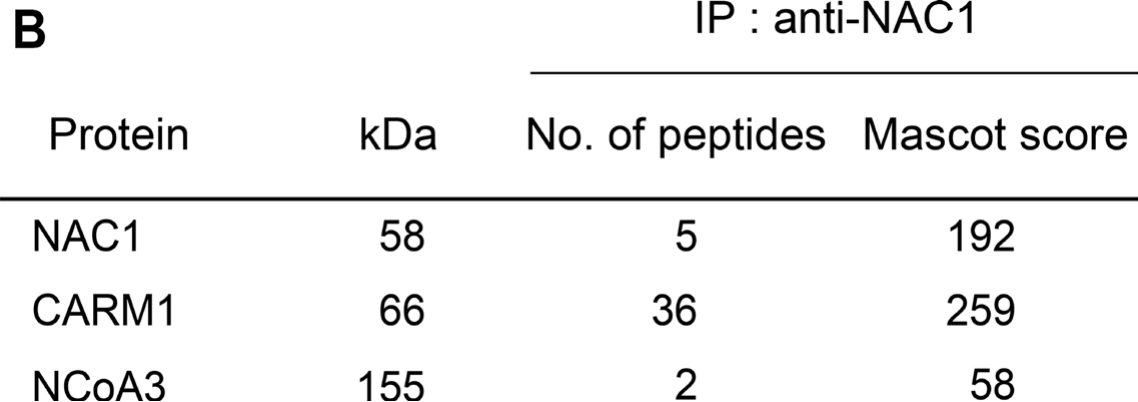

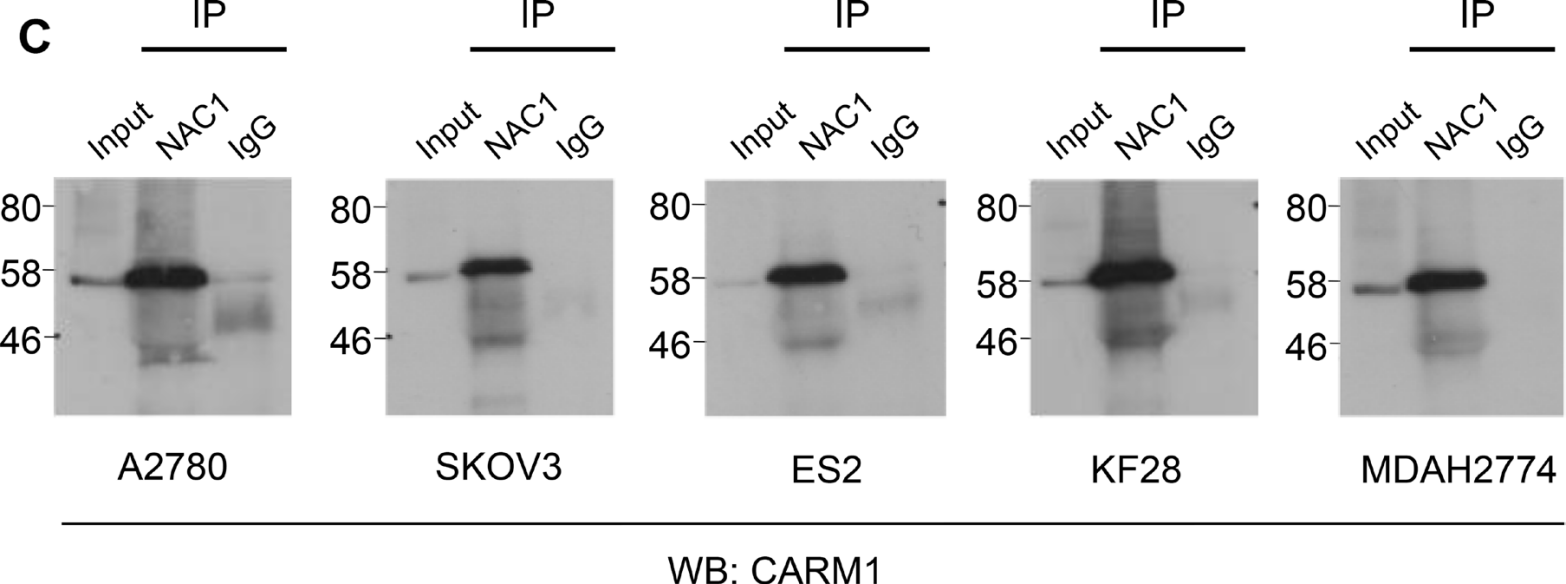

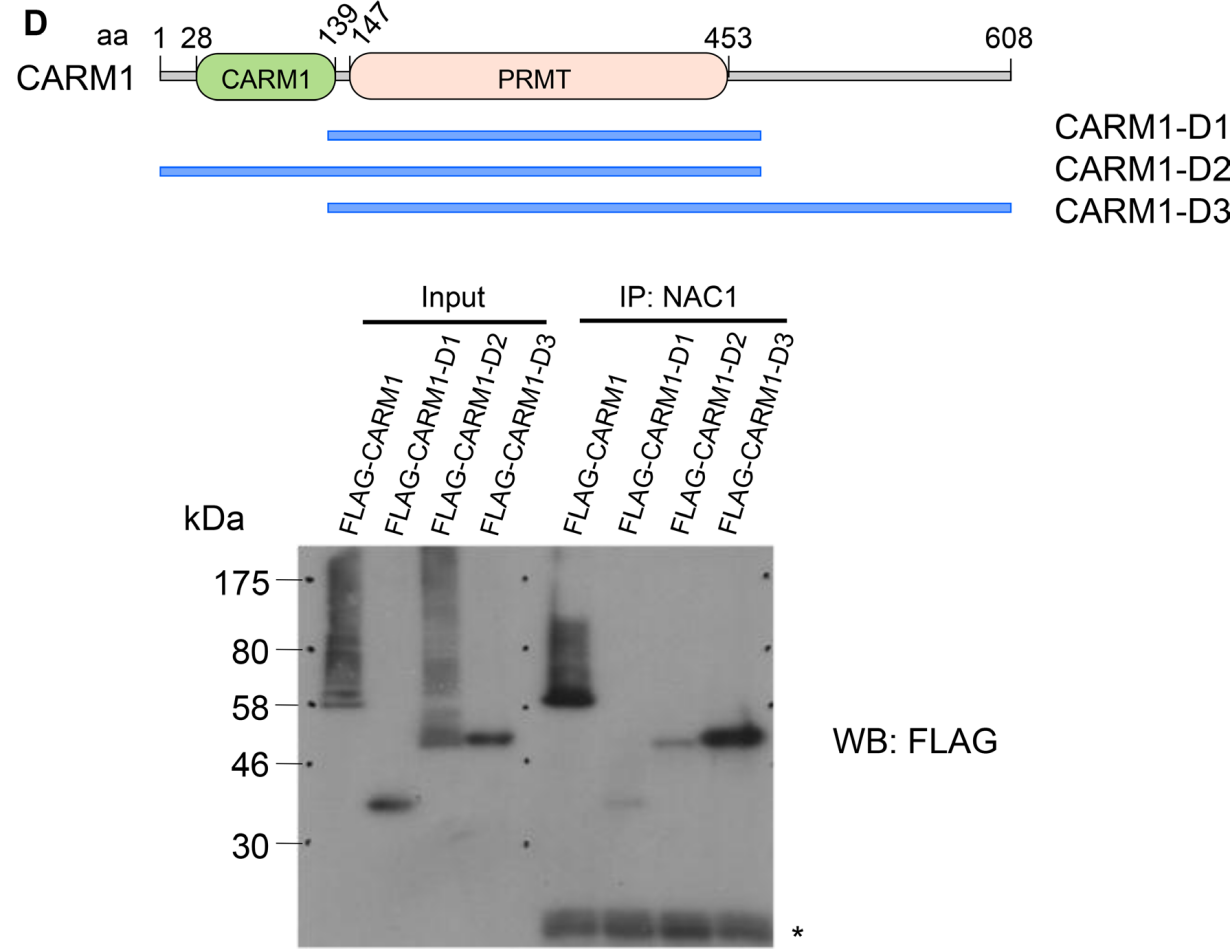

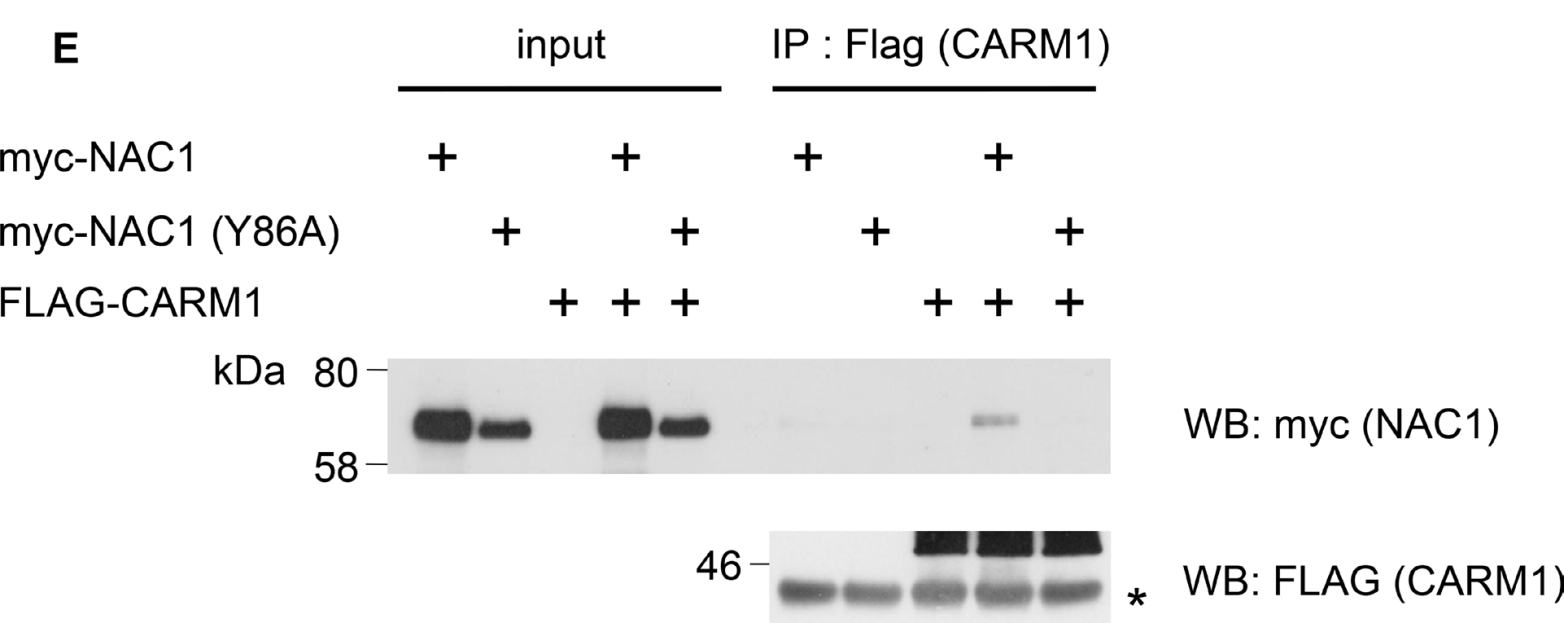
NAC1 interacts with CARM1 in ovarian cancer cells (**A**) Protein extracts of OVCAR3 cells were analyzed by size exclusion chromatography on a FPLC Superdex 200 column. Protein mass standards are indicated above the graph: thyroglobulin (669 kDa), ferritin (440 kDa), catalase (232 kDa), aldolase (158 kDa) and bovine serum albumin (67 kDa). The eluted fractions were analyzed by Western blotting (WB) with the indicated antibodies. (**B**) Mass spectrometry analysis of NAC1, CARM1 and NCoA3 peptides after purification of NAC1-associated proteins. (**C**) Cell lysates of the human ovarian cancer cell lines A2780, SKOV3, ES2, KF28 and MDAH2774 were immunoprecipitated (IP) and Western blotted (WB) with the indicated antibodies. (**D**) A schematic drawing of different truncations of CARM1. Wild-type or mutant CARM1 (D1: 135-483, PRMT; D2: 1-483, ΔC-terminal; or D3: 135-585, ΔCARM1) was expressed in HEK293T cells, immunoprecipitated with anti-NAC1 antibody, and analyzed by Western blotting. aa, amino acids. ^*^, IgL. (**E**) HEK293T cells were transfected with wild-type or mutated form (Y86A) of myc-tagged NAC1, together with FLAG-tagged CARM1. After 48 h, cells were harvested, and immunoprecipitations were performed with anti-FLAG antibody. The precipitates were analyzed by immunoblot probed with the indicated antibodies. ^*^, IgH.

We have recently shown that NAC1 forms 300–500 kDa protein complexes in HeLa human cervical carcinoma cell line [25], even though the estimated molecular mass of human NAC1 protein is 58 kDa. To investigate whether NAC1 forms a protein complex or complexes in ovarian cancer cells, we fractionated 1% (v/v) Triton X-100 ovarian carcinoma OVCAR3 cell lysates according to mass by FPLC using a Superdex 200 sizing column. Immunoblot analysis clearly showed that endogenous NAC1 of OVCAR3 cells eluted as a major peak (fractions 18 to 21) on the Superdex 200 column (Figure 1A), corresponding to a calculated molecular mass of 300–500 kDa, like endogenous NAC1 of HeLa cells.

To understand the transcriptional regulation of NAC1 in cancer progression, we sought to identify proteins that interact with NAC1 in cancer cells. We immunoprecipitated NAC1 complex from the FPLC peak (fractions 18 to 20) of HeLa cells, and then subjected the precipitate to LC-MS/MS analysis. The analysis revealed coactivator-associated arginine methyltransferase 1 (CARM1, also known as protein arginine N-methyltransferase 4, PRMT4) as a major interacting partner of NAC1 (Figure 1B). Notably, a CARM1-interacting protein NCoA3 (nuclear receptor coactivator 3, also known as AIB1, SRC3, p/CIP, ACTR, TRAM-1, and RAC3) was co-purified with NAC1 [26, 27]. The NAC1–CARM1 interaction was confirmed by immunoprecipitation using ovarian cancer cells isolated from several tumors (Figure 1C). Furthermore, CARM1 and NAC1 coelute in the same FPLC fractions of OVCAR3 cell lysates (Figure 1A). To determine the regions within CARM1 that associate with NAC1, we expressed FLAG-tagged full-length or truncated mutants of CARM1 in HEK293T cells. NAC1 interacted with wild-type or mutant CARM1 lacking the amino-terminal domain, but not with CARM1 lacking the carboxy-terminal domain (Figure 1D). These findings demonstrated that the NAC1 protein complex in cancer cells contains CARM1.

The BTB domain of NAC1 mediates homodimerization or heterodimerization with Miz1 [7, 8] and the intranuclear mobility of NAC1 correlated with dimer formation in HeLa cells [25]. We therefore addressed how dimer formation by NAC1 contributes to its binding to CARM1. We utilized the Y86A point mutant of NAC1, which does not form dimers [24]. FLAG-tagged CARM1 were cotransfected with myc-tagged NAC1 or NAC1 (Y86A) into HEK293T cells followed by FLAG immunoprecipitations. NAC1, but not NAC1 (Y86A) was found to co-precipitate with CARM1 (Figure 1E), indicating that dimer formation is committed to the binding of NAC1 to CARM1.

**Figure 2:**
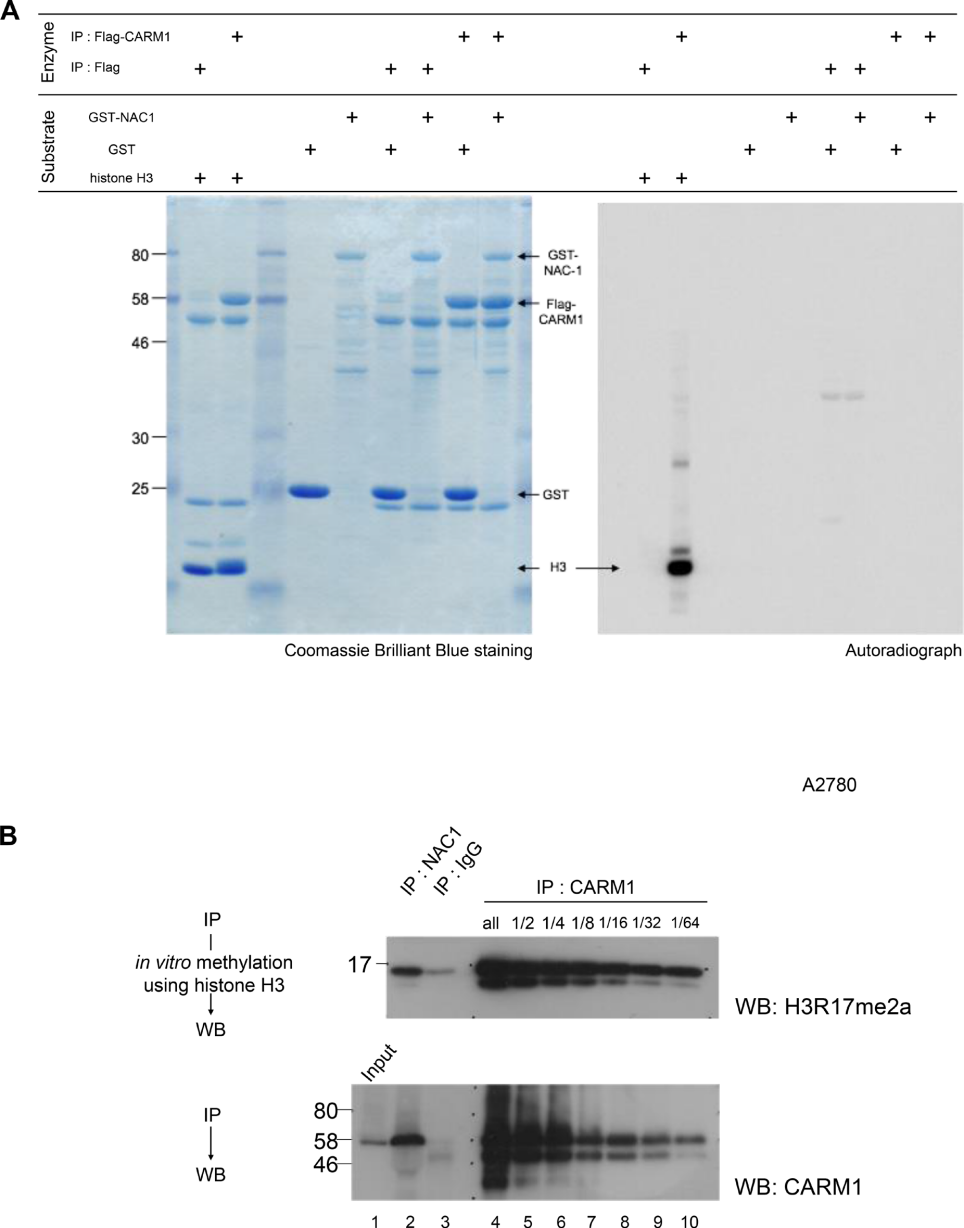
Histone H3, but not NAC1, is methylated by CARM1 associated with NAC1 (**A**) Flag or Flag-CARM1 was expressed in HEK293T cells and the Flag-immunoprecipitates were then used in an *in vitro* methylation reaction with *S*-[methyl-^14^C]-adenosyl-L-methionine (Perkin-Elmer). Recombinant human histone H3, GST or GST-NAC1 proteins were used as substrates. (**B**) *In vitro* methylation reactions were performed using recombinant human histone H3 in the presence of *S*-adenosylmethionine. CARM1 co-immunoprecipitated with NAC1 and CARM1-immunoprecipites of serial dilutions of A2780 cell lysates were used as methyltransferases. Reaction mixtures were separated by SDS-PAGE and Western blotted with anti-histone H3R17me2a (*upper panel*). Immunoprecipitated CARM1 was evaluated by Western blotting with anti-CARM1 (*lower panel*).

CARM1 is a protein arginine N-methyltransferase (PRMT) enzyme that is known to methylate histone H3 (H3R17me2a and H3R26me2a), and nonhistone substrates [29]. We therefore performed *in vitro* methylation assays where CARM1 immunoprecipitated from HEK293T cells was incubated with recombinant histone H3, GST or GST-NAC1 proteins as substrates and with [^14^C]SAM as a methyl donor. The analysis clearly showed that CARM1 methylates histone H3, but not GST or GST-NAC1 (Figure 2A). To determine whether the association of NAC1 influences the methyltransferase activity of CARM1, CARM1 co-immunoprecipitated with the endogenous NAC1 of A2780 cells was subjected to an *in vitro* methylation assay with histone H3 and the reaction products were examined by Western blotting with anti-histone H3R17me2a. The analysis showed that CARM1 bound to NAC1 methylates Arg 17 of histone H3 compared to an equal amount of immunoprecipitated CARM1 (Figure 2B, compare lanes 2 and 8), revealing that CARM1 methylates histone H3 even in association with NAC1.

### Positive correlation between the expression of NAC1 and CARM1 in ovarian carcinoma tissues

**Figure 3:**
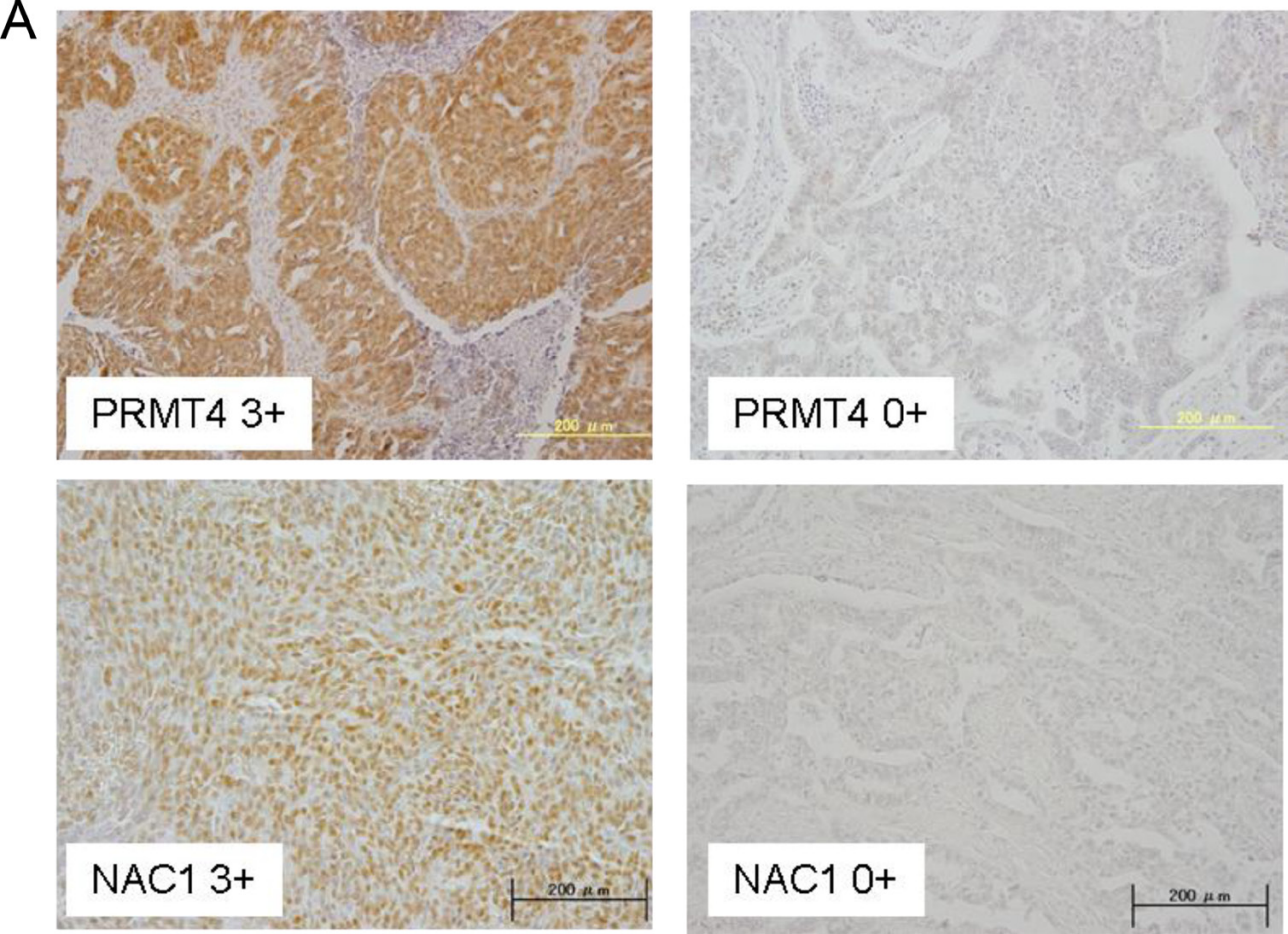

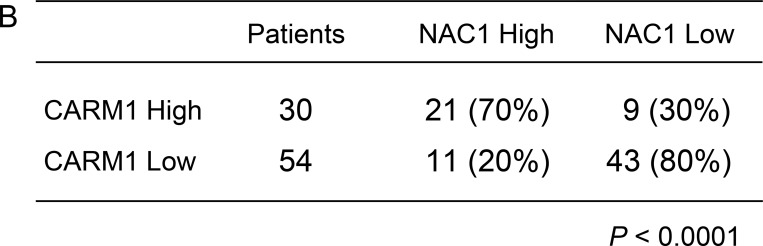

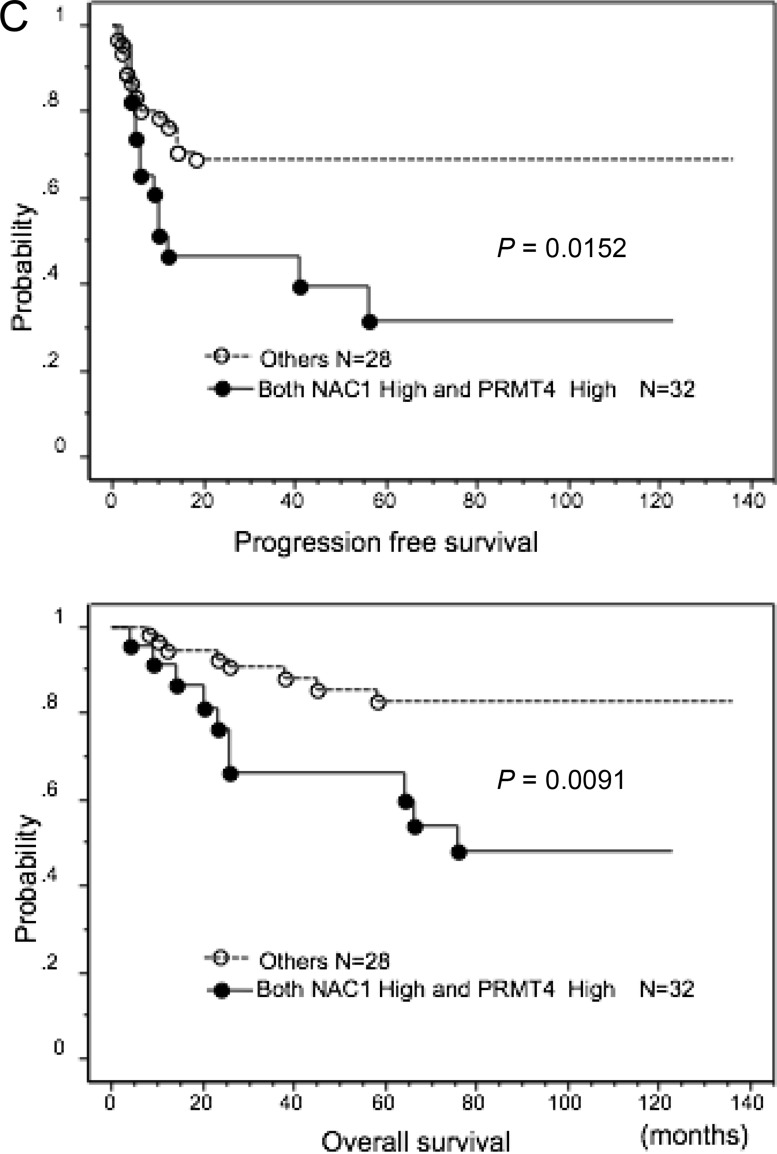

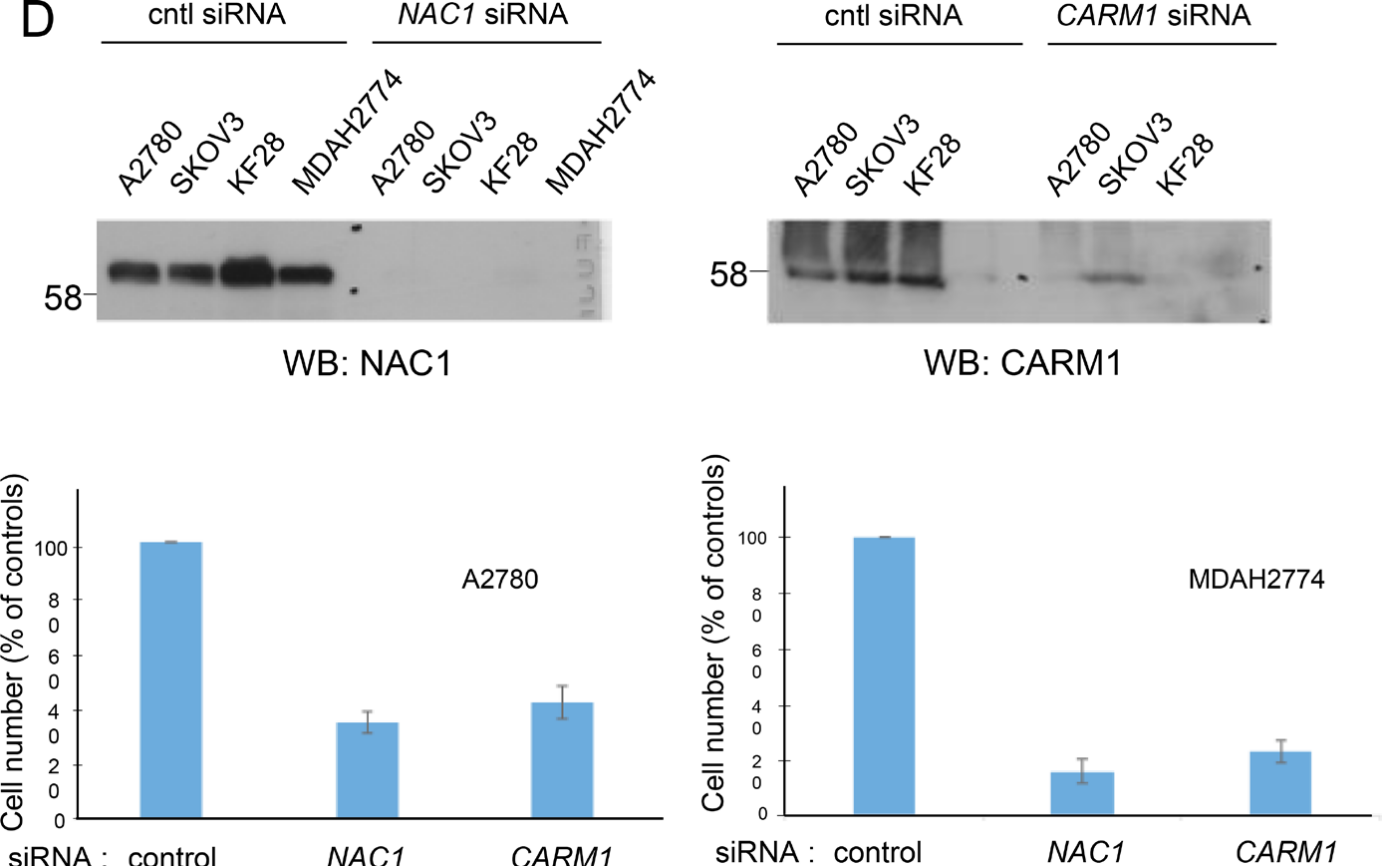
Co-upregulation of CARM1 and NAC1 in ovarian carcinoma tissues (**A**) Immunoreactivities of CARM1 and NAC1 in ovarian carcinoma tissues. Intense CARM1 immunoreactivity is present in the nuclei and cytoplasm of ovarian carcinoma cells (*upper left panel*: CARM1 3+). Intense NAC1 immunoreactivity is present in the nuclei of ovarian carcinoma cells (*lower left panel*: NAC1 3+). An ovarian carcinoma case with negative staining for CARM1 (CARM1 0+) and NAC1 (NAC1 0+). (**B**) The relationship between the expression of CARM1 and NAC1. *P* < 0.0001; calculated with Pearson’s chi-square test. (**C**) Kaplan–Meier curves with log-rank test for progression-free (*upper panel*) and overall (*lower panel*) survival according to NAC1 and CARM1 status. (**D**) Effects of NAC1 and CARM1 knockdown in ovarian cancer cells. Western blotting of NAC1 (*upper left panel*) and CARM1 (*upper right panel*) in indicated siRNA-treated ovarian cancer cells. *Lower panel*: cell proliferation of control, *NAC1* or *CARM1* siRNA-treated ovarian cancer cells A2780 (*lower left panel*) and MDAH2774 (*lower right panel*). Columns, mean (*n* = 3); bars, SD.

In an attempt to determine the expression levels of CARM1 and NAC1 in clinical specimens, we performed immunohistochemistry (IHC) for CARM1 and NAC1 in 84 ovarian carcinoma tissues. Both antibodies stained tumors more strongly than the surrounding benign tissues (Figure 3A). High expression of NAC1 and CARM1 (> immunoreactivity 2+ and 3+) was observed in 38.1% (32/84) and 35.7% (30/84) of the analyzed tumors, respectively. Figure 3B shows a positive correlation between NAC1 and CARM1 immunoreactivities, with a chi-squared test result of *P* < 0.0001. The significant correlation of CARM1 and NAC1 expression levels implies that NAC1 functions synergistically with at least CARM1 to promote tumorigenesis in ovarian cancers.

Up-regulation of NAC1 in human carcinomas was shown to confer resistance to the chemotherapeutic drug paclitaxel as well as to contribute to tumor growth and poor survival [15, 16]. When patients with ovarian carcinoma treated with platinum-based chemotherapy were classified using a two-tier system of expression levels (low or high), the log-rank test revealed that progression-free and overall survival were shorter in patients with highly expressed NAC1 and CARM1 than in those with low expressed proteins (*P* = 0.0152 and *P* = 0.0091, respectively) (Figure 3C). Univariate analysis demonstrated that International Federation of Gynecology and Obstetrics (FIGO) stages III and IV (*P* < 0.0001, *P* = 0.034; log-rank test), residual tumor ≥1 cm (*P* < 0.0001, *P* = 0.0012; log-rank test), and high NAC1/CARM1 expression (*P* = 0.0152, *P* = 0.0091; log-rank test) correlated with shorter progression-free and overall survival (Tables 1 and 2). When data were stratified for multivariate analysis, only residual tumor remained significant (*P* = 0.0002) for shorter progression-free survival (Table 1), but residual tumor (≥1 cm) and high NAC1/CARM1 expression remained significant (*P* = 0.0027 and *P* = 0.0418, respectively) for overall survival (Table 2). These results suggest that high expression levels of NAC1 and CARM1 may serve as a prognostic biomarker for predicting resistance to chemotherapy.

**Table 1:** Univariate and multivariate analyses of progression-free prognostic factors in patients with ovarian cancer

Factors	Patients	Univariate			Multivariate		
		hazard ratio	95% CI	*P* value	hazard ratio	95% CI	*P* value
FIGO stage							
III, IV	44	6.8	2.6–17.7	< 0.0001	1.5	0.7–6.4	0.1903
I, II	40						
Grade							
G2, G3	67	2	0.7–5.8	0.1838	NA	NA	NA
G1	17						
Histology							
Serous	44	2	0.7–5.8	0.8	NA	NA	NA
Others	40						
Age (years)							
<60	42	1.7	0.8–3.4	0.1527	NA	NA	NA
≧60	42						
Residual tumor							
≧1 cm	37	11.7	4.4–30.8	< 0.0001	7.8	2.6–23.3	0.0002
<1 cm	47						
NAC1/PRMT4 immunostaining							
NAC1 High /PRMT4 High	23	2.4	1.2–4.8	0.0152	1.8	0.9–3.7	0.1149
Others	61						

**Table 2:** Univariate and multivariate analyses of overall prognostic factors in patients with ovarian cancer

Factors	Patients	Univariate			Multivariate		
		hazard ratio	95% CI	*P* value	hazard ratio	95% CI	*P* value
FIGO stage							
III, IV	44	3.3	1.1–10.1	0.0334	0.6	0.1–2.4	0.4573
I, II	40						
Grade							
G2, G3	67	4.1	0.5–31.1	0.169	NA	NA	NA
G1	17						
Histology							
Serous	44	2	0.7–5.8	0.8	NA	NA	NA
Others	40						
Age (years)							
<60	42	1	0.4–2.5	0.994	NA	NA	NA
≧60	42						
Residual tumor							
≧1 cm	37	11.4	2.6–49.8	0.0012	13.1	2.4–70.1	0.0027
<1 cm	47						
NAC1/PRMT4 immunostaining							
NAC1 High /PRMT4 High	23	3.5	1.4–8.8	0.0091	3	1.0–8.4	0.0418
Others	61						

The knockdown of CARM1 as well as NAC1 using siRNA suppressed cell growth in the ovarian carcinoma cell lines A2780 and MDAH2774 (Figure 3D).

## DISCUSSION

The present study indicates that NAC1 forms 300–500 kDa protein complexes in ovarian cancer cells, comparable with that of HeLa cells [25]. The most striking finding of this study lies in the identification of CARM1 as a protein interacting with NAC1 in a protein complex. Tissue microarray analysis revealed significant correlation of CARM1 and NAC1 expression levels. Furthermore, ovarian cancer patients expressing high levels of NAC1 and CARM1 exhibited poor prognosis after adjuvant chemotherapy (Figure 3C). Thus, high expression levels of NAC1 and CARM1 may serve as an informative prognostic biomarker for predicting resistance to chemotherapy for ovarian cancer.

The present study reveals that NAC1 associates with CARM1 in a 300–500 kDa protein complex in ovarian cancer cells (Figure 1). CARM1 has been implicated in cancer. High-level expression of CARM1 has been observed in several cancers, including those of prostate [29, 30], colon [30], and breast [29–33], with levels higher in metastatic breast cancer than in primary breast cancer [33]. Kim *et al.* reported that CARM1 overexpression was noted only in a small number (17%) of ovarian cancer patients [30]. CARM1 has also been shown to stimulate cancer growth [34] and serves coactivator roles for numerous proteins that have been implicated in cancer, including p53, E2F1, cyclin E1, NF-κB, and steroid hormone nuclear receptors (reviewed [35, 36]). In agreement with these previous studies, we showed that CARM1 is overexpressed in human ovarian cancers (30/84, 35.7%), with significant correlation of high NAC1 expression levels, and elevated levels of NAC1 and CARM1 correlate with poor prognosis after adjuvant chemotherapy (Figure 3C). In accordance with the patient findings, knockdown of NAC1 and CARM1 in ovarian cancer cell lines resulted in cell growth inhibition (Figure 3D). Thus, CARM1 may play a role in ovarian cancer progression in collaboration with NAC1, and high expression levels of NAC1 and CARM1 may serve as an informative prognostic biomarker for predicting resistance to chemotherapy for ovarian cancer. Based on the concept of ‘oncogene addiction’ [37], CARM1 may represent a novel therapeutic target in ovarian cancer.

There is growing evidence that histone methylations by PRMTs have an important aspect for the dynamic regulation of gene expression [28, 35, 36]. CARM1 is a protein arginine N-methyltransferase that catalyzes the formation of asymmetric dimethylarginine. CARM1 initially was described as a transcriptional activator of the p160 histone acetyltransferase family of nuclear receptor-associated proteins [26]. We demonstrated that the NAC1-binding region of CARM1 locates at the C-terminal part outside of N-terminal catalytic domain (Figure 1D) and that the interaction does not disrupt the catalytic function of CARM1 (Figure 2B). This implied that NAC1 is a gene-specific transcription factor and the interacting partner CARM1 functions as a coactivator together with the p160 family of nuclear receptor-associated proteins in cancer cells. Indeed, we identified NCoA3, one of the p160 family in NAC1 immunoprecipitate followed by LC-MS/MS analysis (Figure 1B). Even though we could not validate if NAC1 immnoprecipitated NCOA3 in several ovarian cancer cell lines due to the quality of anti-NCOA3 antibody which could not detect endogenous NCOA3 expression, it is highly possible that NAC1 formed protein complex with CARM1 and NCOA3. It is therefore important to precisely identify the specific gene sets that NAC1 selects as a transcription factor and to elucidate how NAC1 recognizes the gene sets in cancer cells.

In neuronal cells, NAC1 is known to interact with the histone deacetylases HDAC3 (49 kDa) and HDAC4 (119 kDa) [38], and with REST corepressor 1 (CoREST, 53 kDa) [39], but not with other corepressors (nuclear receptor corepressor 1 (NCoR), nuclear receptor corepressor 2 (NCoR2, also known as SMRT), or mSin3a) [38]. In the present LC-MS/MS analysis we did not detect these corepressors in the NAC1 complex isolated from cancer cells. CARM1 plays important role in ER (estrogen receptor) signaling pathway [31–33]. H3R17me2a epigenetic modification is the hall mark of this pathway and catalyzed by CARM1 in corporation with PAF1c [40]. We did not address the relationship of NAC1 to this pathway since PAF1c was not detected in protein complex. However, this should be examined in further study.

Dimerization is frequently observed in transcription factors [41]. Recent studies have shown that the BTB domain of NAC1 mediates homodimerization or heterodimerization with Miz1 [7, 8] and that the intranuclear mobility of NAC1 correlated with dimer formation in HeLa cells [25]. By use of the Y86A mutant of NAC1 with resistance to dimer formation [23], this study reveals that dimerization is a prerequisite for its binding to CARM1 (Figure 1E). The Y86 was oriented to the dimerization interface [7] and the point mutant Y86A is a powerful tool with which we study how dimer formation contributes to the biological functions of NAC1. By analyzing a series of CARM1 deletion mutants, we first identified the C-terminal part as the binding region with NAC1 (Figure 1D). We then tried to determine the binding region of NAC1 with CARM1, but both N-terminal (1-250, harboring BTB domain and NLS) and C-terminal (251-527, harboring BEN domain) half of NAC1 did not bind CARM1 in coexpression system (data not shown). The result supports that both of dimerization and C-terminal half of NAC1 is essential for its binding to CARM1.

NAC1 is known to be important for the pluripotency of embryonic stem (ES) cells [42, 43], and was shown to promote mesendodermal and repress neuroectodermal fate selection in ES cells, in cooperation with two other pluripotency transcription factors, Oct4 and Sox2 [44]. CARM1 was also shown to be required for the self-renewal and pluripotency of ES cells, and to play its role, at least in part, by sustaining *Oct4* and *Sox2* activity through arginine methylation of histone H3 at their promoters [45]. Taken together with these previous reports, the present study infers the existence of a close functional coupling between NAC1 and CARM1 in ES cells.

In summary, while NAC1 is currently considered to act as a transcription suppressor based on previous studies focusing on neuronal cells, the present study showed that NAC1 may act as a transcriptional activator to fulfill the oncogenic potential in ovarian cancer cells in cooperation with the interacting partner CARM1. The results reported here may have significant implications for future studies aimed at elucidating the pathogenesis of ovarian cancer.

## MATERIALS AND METHODS

### Antibodies

Monoclonal anti-NAC1 (9.27) antibody has been described [23]. The following commercial antibodies were used: polyclonal anti-CARM1 (A300-421A, Bethyl Laboratories, TX, USA); anti-FLAG (60-031, BioAcademia, Osaka, Japan); anti-histone H3 dimethyl Arg17 (H3R17me2a, asymmetric) (39709, Active Motif, Carlsbad, CA, USA); horseradish peroxidase-conjugated goat F(ab’)2 anti-mouse (710-1332, Rockland Immunochemicals, Limerick, PA, USA) and goat anti-rabbit IgG (111-035-003, Jackson ImmunoResearch Laboratories, West Grove, PA, USA).

### Cell lines

The human ovarian serous carcinoma cell lines OVCAR3, A2780, SKOV3, ES2 and MDAH2774 were obtained from the American Tissue Culture Center. The HeLa human cervical epitheloid carcinoma cell line (JCRB9004) was purchased from the Japanese Collection of Research Bioresources Cell Bank. Cell line characterization and authentication were performed using morphology, karyotyping, PCR, and STR profile by the cell banks. The KF28 human ovarian serous carcinoma cell line was a kind gift from Dr. Yoshihiro Kikuchi (Ohki Memorial Kikuchi Cancer Clinic for Women) [24]. The cell line was authenticated by the supplier. To maintain authenticity of the cell lines, multiple aliquots of frozen stocks were prepared from initial stocks, and every 3 months, a new frozen stock was used for the experiments. The cells were routinely inspected for identity by morphology and growth curve analysis and validated to be *mycoplasma* free.

### Plasmid construction

Human histone H3 and CARM1 full-length cDNAs obtained by reverse transcribed PCR using the total RNA from HeLa cells were cloned into pMXs-FHG and Flag/pcDNA3, respectively [23]. Fragments of the human CARM1 gene encoding residues 135–483 (CARM1-D1), 2-483 (CARM1-D2, removal of the first methionine) and 135–608 (CARM1-D3) were cloned into Flag/pcDNA3 by PCR using CARM1 full-length cDNA as template. All PCR-amplified cDNA products were fully sequenced using a 3130 genetic analyzer (Thermo Fisher Scientific) to confirm the sequences and to verify the absence of secondary point mutations.

### Silencing RNA knockdown of *NAC1* and *CARM1* gene expression

Stealth small interfering RNA (siRNA) against *NAC1* (#1, 5′-CCGGCUGAACUUAUCAACCAGAUUG-3′) [22] and *CARM1* (#1, 5′-CACCCUUCACGGAUGAACAGCUCUA-3′; #2, 5′-CCAAGUCCAGUAACCUCCUGGAUCU-3′) were purchased from Thermo Fisher Scientific. Cells were seeded on 96-well plates and transfected with siRNAs using RNAiMAX (Thermo Fisher Scientific) according to the manufacturer’s instructions. The cell number was determined indirectly by an MTT assay 72 hours after the transfection with siRNA. Data are shown as the means ± 1 standard deviation of triplicate determinations.

### Protein purification

Triton X-100 (1%, v/v) lysate of HeLa cells was fractionated by fast protein liquid chromatography (FPLC) on a Superdex 200 Increase 10/300 GL sizing column (GE Healthcare, Buckinghamshire, UK) and eluted with phosphate-buffered saline containing Triton X-100 (1%, v/v). Fractions (0.5 ml) were collected and proteins were resolved by SDS-PAGE. NAC1 was detected on immunoblots with anti-NAC1 monoclonal antibody 9.27 [22]. FPLC fractions 18 to 20 were immunoprecipitated with anti-NAC1 antibody, resolved on a SDS-polyacrylamide gel, and stained using SilverQuest (Thermo Fisher Scientific). Each specific polypeptide band was excised, destained, and trypsinized for liquid chromatography-tandem mass spectrometry (LC-MS/MS) analysis. Data from each LC-MS/MS analysis were assembled and analyzed using the proteome software Scaffold (Proteome Software), and the number of assigned spectra and the score obtained from Mascot searches are summarized in Figure 1B.

### *In vitro* methylation assay

Reaction mixture (30 µl) containing 1 µg of different recombinant human histone H3, GST and GST-NAC1 (as substrates), the indicated immunoprecipitate (as enzyme), and 1 µL of *S*-[methyl-^14^C]-adenosyl-L-methionine (0.02 mCi/mL, Perkin-Elmer, Waltham, MA) (for hot assay) or 20 µM *S*-adenosylmethionine (SAM) (for cold assay) in methylase activity buffer (50 mM Tris, pH 8.5, 100 mM NaCl, 1 mM dithiothreitol) was incubated for 60 min at 30° C. The reaction products were separated by 15% SDS-polyacrylamide gel electrophoresis, followed by Coomassie Brilliant Blue staining and autoradiography (for hot assay) or by Western blotting with anti-histone H3R17me2a (for cold assay).

### Tissue samples

Formalin-fixed, paraffin-embedded tissue samples of 84 ovarian cancers, including 44 serous carcinomas, 10 mucinous carcinomas, 10 clear cell carcinomas, and 20 endometrioid carcinomas, were used in the present study. Diagnoses were based on conventional morphological examinations of sections stained with hematoxylin and eosin (H&E), and tumors were classified according to the WHO classification. Tumor staging was performed according to the International Federation of Gynecology and Obstetrics (FIGO) classification. Samples were obtained from the Department of Obstetrics and Gynecology at Shimane University Hospital. All patients were primarily treated with cytoreductive surgery and adjuvant platinum and taxane chemotherapy (CBDCA AUC5 with paclitaxel 175 mg/m^2^ or docetaxel 70 mg/m^2^). All patients received 6-12 courses of this combination regimen. The Shimane University Institutional Review Board approved the acquisition of tumor tissues and written informed consent was obtained from all subjects.

### Immunohistochemistry

Paraffin-embedded tissues were organized into tissue microarrays, made by removing tumor cores 3 mm in diameter from each block. Selection of the area corresponding to the core was made by a gynecological oncologist (K. N.) and pathology technician (K. I.) and was based on a review of H&E slides. The immunohistochemistry method and the evaluation criteria were detailed in a previous study [15].

### Statistical analysis

Progression-free and overall survivals were calculated from the date of diagnosis to the date of first relapse or last follow-up. Age and performance status distributions were similar between patients expressing and not expressing NAC1 or CARM1. Data were plotted as Kaplan–Meier curves, and the significance of differences was determined using the log-rank test. A multivariate prognostic analysis was performed using a Cox proportional hazards model. Data were censored when patients were lost to the follow-up. Student’s *t*-test was used to examine the significance of differences in growth assay data. Chi-squared test or Fischer’s exact test was used for comparisons of categorical data. Data are presented as means ± SD. *P* < 0.05 was considered significant.
